# Psychometric comparison of two short versions of the Perceived Stress Scale (PSS-4) in a representative sample of the German population

**DOI:** 10.3389/fpsyg.2024.1479701

**Published:** 2025-01-06

**Authors:** Bjarne Schmalbach, Mareike Ernst, Elmar Brähler, Katja Petrowski

**Affiliations:** ^1^Medical Psychology and Medical Sociology, University Medical Center of the Johannes Gutenberg-University Mainz, Mainz, Germany; ^2^Department of Clinical Psychology, Psychotherapy and Psychoanalysis, Institute of Psychology, University of Klagenfurt, Klagenfurt am Wörthersee, Austria; ^3^Department of Psychosomatic Medicine and Psychotherapy, University Medical Center Mainz, Johannes Gutenberg University Mainz, Mainz, Germany; ^4^Integrated Research and Treatment Center Adiposity Diseases, Behavioral Medicine Unit, Department of Psychosomatic Medicine and Psychotherapy, Leipzig University Medical Center, Leipzig, Germany

**Keywords:** stress, measurement instrument, psychometric analysis, screening instrument, factor analysis

## Abstract

Perceived stress is a construct of crucial importance to health and well-being, necessitating the provision of economic, psychometrically sound instruments to assess it in routine clinical practice and large-scale survey studies. Two competing short versions of the Perceived Stress Scale (PSS), each consisting of four items, have been proposed. In the present study, we compare the two in a sample representative of the German general population (*n* = 2,527). Our analyses show that both versions are sufficiently reliable and valid, given the right measurement model. Specifically, the original PSS-4 by Cohen et al. suffers from response style effects, which we remedied using random intercept factor analysis. With the addition of the method factor, it is a highly reliable and valid scale. The PSS-2&2 by Schäfer et al. is more complex in its interpretation since it is split into two facets which cannot be summarized into a single score. Specifically, the Helplessness subscale correlates with related constructs very similar to the original unifactorial model but its reliability is lackluster. In contrast, the Self-Efficacy subscale is reliable but diverges in terms of its correlational pattern. In sum, both versions can be recommended for research designs in need of a brief measure of stress and offer unique contributions.

## 1 Introduction

Acute and chronic psychological stress has long been identified as a crucial determinant of physical and psychological health as well as overall well-being (Cohen et al., 2007; Kivimäki and Steptoe, 2018; O’Connor et al., 2021; Wirtz and von Känel, 2017). The stress response is an evolutionarily adaptive mechanism designed to enable individuals to cope with acute threats, often referred to as the “fight-or-flight” response. When confronted with a stressor, the body activates the hypothalamic–pituitary–adrenal (HPA) axis and the sympathetic nervous system, leading to the release of stress hormones, such as cortisol and adrenaline. These physiological changes increase heart rate, sharpen attention, and mobilize energy resources, which together enhance the organism’s ability to respond quickly and effectively to immediate challenges. However, when stress becomes chronic—whether through ongoing or repeated exposure to stressors or inadequate/insufficient coping strategies, or a combination of the two—the body remains in a state of heightened arousal, which can have a range of negative physical and mental health consequences (Agorastos and Chrousos, 2022). Prolonged activation of the HPA axis implicates high allostatic load, a cumulative “wear and tear” on the body, resulting in dysregulation, affecting immune function, cardiovascular health, and metabolic processes. This chronic stress response has been linked to diverse adverse events, including increased risks for cardiovascular disease, hypertension, and depression, as well as cognitive impairments due to neural damage in stress-sensitive areas like the hippocampus (McEwen, 2017). It can therefore be assumed that stress plays an important role in the incidence and chronicity of widespread population diseases.

Given this substantial impact of stress on health, its valid and reliable measurement is of great importance in many research questions across various settings, comprising clinical as well as population-level investigations (Crosswell and Lockwood, 2020; Epel et al., 2018; Giannakakis et al., 2019). Measuring stress accurately allows for targeted prevention and intervention efforts. The fact that the most influential psychological models define stress as the result of an individual’s appraisal of a situation supports the use of self-report measures which provide insights into the subjective experience: According to the transactional model of stress and coping, stress is not only a physiological response but also a subjective experience shaped by the individual appraisals of stressors. Lazarus and Folkman (1984) emphasized that stress arises when a person perceives a situation as threatening or demanding, exceeding their coping resources. This cognitive appraisal process affects how often and intensely the stress response is activated, directly influencing the above-described physiological outcomes and contributing to allostatic load over time.

The Perceived Stress Scale (PSS; Cohen et al., 1983; Cohen and Williamson, 1988) is one of the most widely applied measures of stress, and two competing ultra-short versions have been suggested by previous research. First, the original authors (Cohen et al., 1983) of the PSS suggested the configuration marked in Supplementary Table S1. This version, however, has been shown repeatedly to suffer from suboptimal factorial validity (Demkowicz et al., 2020; Ingram IV et al., 2016; Mondo et al., 2021). Recently, Schäfer et al. (2023) proposed an alternative version which includes two correlated factors, Helplessness and Self-Efficacy, captured using the items marked in Supplementary Table S1 – but does not yield a total score.

The present study aims to test both versions in a representative sample of the German population and compare them with regard to their psychometric merits. Specifically, we will test whether the multi-dimensionality introduced by Schäfer et al. (2023) is necessary and reflects actual properties of the latent stress construct or whether it is just a method artefact caused by response biases commonly encountered with reverse-coded items: acquiescence (Maydeu-Olivares and Coffman, 2006; Podsakoff et al., 2003).

## 2 Method

### 2.1 Participants and procedure

The present survey sample was collected in 2014 by a German market research agency (Unabhängiger Service für Umfragen, Methoden und Analysen, Berlin, Germany). The ethics committee of the University of Leipzig approved the present investigation (063-14-10032014). To obtain a representative survey, a random-route procedure was utilized: First, regions were identified based on electoral districts. Second, within these regions, households were randomly selected. Third, within the household, the respondent was determined based on the Kish selection grid. Out of the 4,607 households that were initially contacted, 55.1% gave their informed consent and participated in the survey. The remaining sample of 2,527 is described in Table 1.

**Table 1:** Sample description.

	*n*	%
Sex		
Female	1,350	53.4
Male	1,177	46.6
Age, *M/SD*	49.44	17.82
Marital status		
Married	1,165	46.1
Separated	53	2.1
Single	688	27.2
Divorced	352	13.9
Widowed	267	10.6
Education		
<10 years	971	38.4
=10 years	994	39.3
>10 years	494	19.5
Currently in school	68	2.7
Employment		
Working full-time (>35 h)	993	39.3
Working part-time (15-35 h)	301	11.9
Working minimal hours or not working (includes home-makers, pensioners, etc.)	1,051	41.6
In school/apprenticeship/university	172	6.8
Household net income		
< 1,500€	716	28.3
< 2,500€	822	32.5
≥ 2,500€	918	36.3
No response	71	2.8

### 2.2 Instruments

The Perceived Stress Scale (PSS-10, Cohen et al., 1983; Schäfer et al., 2023; Schneider et al., 2020) assesses an individual’s acute stress level using 10 items and a 5-point scale. It mainly focuses on the extent to which the respondent feels capable (or incapable) of handling their daily life and upcoming challenges. The German version has previously been investigated regarding its psychometric properties and shown mostly good internal consistency across samples (Reis et al., 2019).

The Personal Burnout Scale (PBS, Nübling et al., 2006; Pejtersen et al., 2010) of the Copenhagen Psychosocial Questionnaire (6 items, *ω* = 0.915 in this sample) was used to measure physical and mental exhaustion. Specifically, it uses a 6-point scale to inquire into the frequency of the following states: tired, physically exhausted, emotionally exhausted, unable to go on, weak and prone to illness.

The Questions on Life Satisfaction (FLZ-8; Henrich and Herschbach, 2000), specifically the General Life Satisfaction Module, is an 8-item instrument that quantifies a respondent’s life satisfaction. It does so using a 5-point response scale. In the current sample, reliability was estimated at *ω* = 0.816.

The Patient Health Questionnaire (PHQ-4; Kroenke et al., 2009; Löwe et al., 2010) is a brief measure of symptoms of depression (PHQ-2) and anxiety (Generalized Anxiety Disorder-2, GAD-2), consisting of two items each. Respondents indicate their agreement with the respective symptom descriptions on a 4-point scale. Reliability in the present sample was ω = 0.763 and 0.778, respectively.

### 2.3 Statistical methods

All analyses of the study at hand were carried out in R (version 4.4.1), using the packages *ezCutoffs*, *lavaan* and *semTools* (Jorgensen et al., 2022; Rosseel, 2012; Schmalbach et al., 2019). Initially, we calculated a congeneric factor analysis model as well as a random intercept model (Maydeu-Olivares and Coffman, 2006) for the Cohen-PSS. Specifically for this model, the method factor loadings for all items are set to equality and the method and content factor are set to not correlate. For the Schäfer-PSS, we calculated a correlated-factors model. To estimate, we utilized the robust full-information maximum likelihood estimator (*MLR*) – although the number of missing values was negligible (0.4%). We then inspected χ^2^, the Comparative Fit Index (*CFI*), the Tucker-Lewis Index (*TLI*), the Root Mean Square Error of Approximation (*RMSEA*), and the Standardized Root Mean Square Residual (*SRMR*). As per recommendations by Schermelleh-Engel et al. (2003), the values for *CFI*/*TLI* should be greater than 0.95 (better yet 0.97), and *RMSEA*/*SRMR* should be smaller than 0.08/0.10 (better yet 0.05). To supplement these fixed cutoffs, we additionally calculated simulated cutoff values using the *ezCutoffs* package using 1,000 replications and an *α* of 0.05. We report McDonald’s *ω* as a measure of internal consistency (Dunn et al., 2014). For the calculation of convergent correlations, we included separate factors for all relevant scales in each of the three PSS models and estimated the interfactor correlations.

## 3 Results

### 3.1 Factorial validity

**Figure 1:**
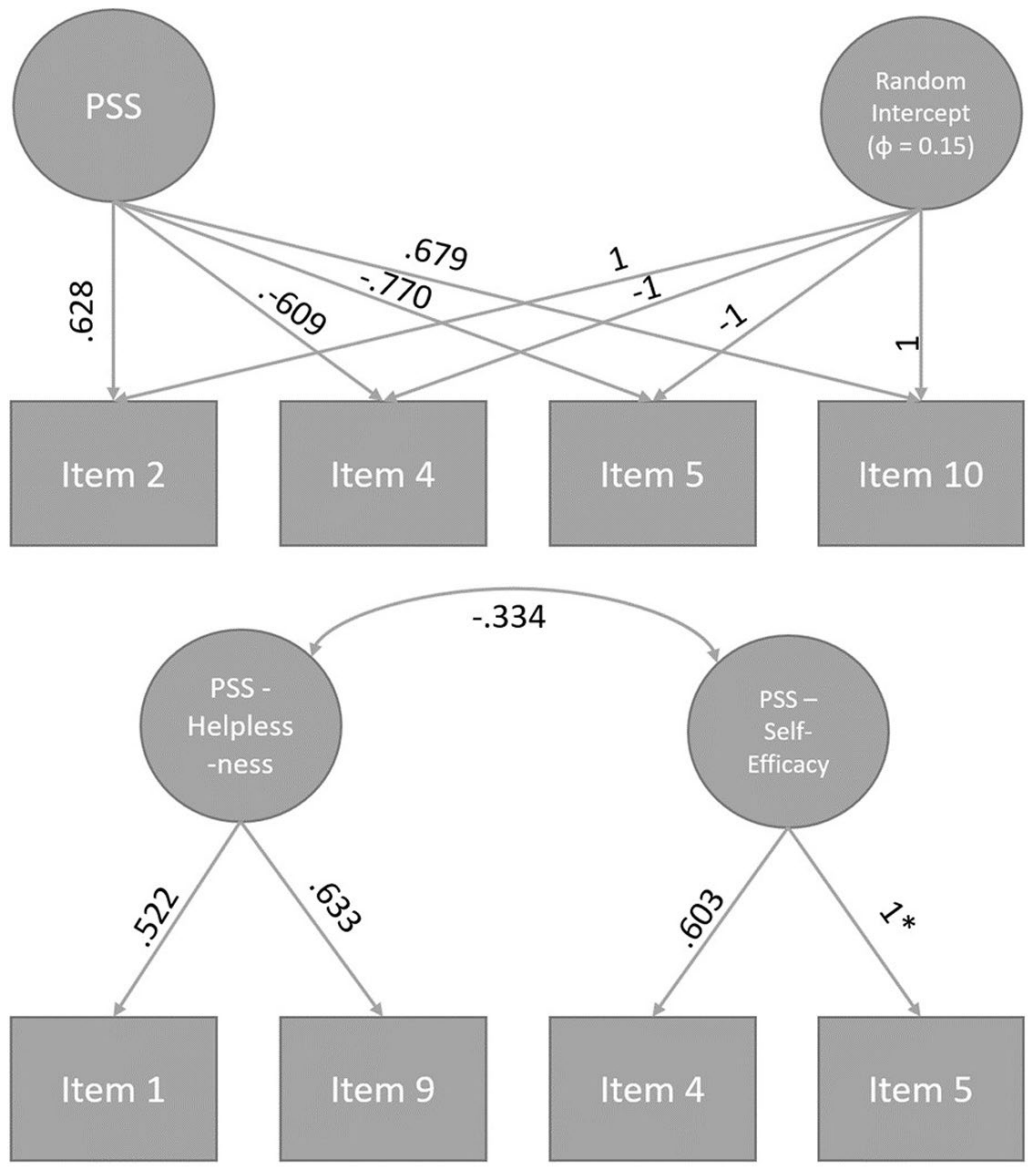
Path diagrams of the final models of the Perceived Stress Scale 4. Models were standardized by setting the latent variable variances to 1 with the exception of the random intercept factor. *This standardized factor loading is equal to one because of the negative error variance of Item 5 which was then set to ≥0.

We present the model fit results from the various factor analyses in Table 2, along with the path diagrams for the final models in Figure 1. In summary, the congeneric model for the Cohen-PSS is unacceptable by all applied standards (except the *SRMR* fixed cutoff), although its reliability was acceptable at ω = 0.71. Including a method factor for acquiescence dramatically improved model fit – both in terms of traditional fixed and simulated cutoffs. Reliability of the content factor also improved, ω = 0.82.

**Table 2:** Fit results for the various PSS-4 models.

Model	χ^2^(df)	*p*	CFI	TLI	RMSEA	SRMR
Cohen-PSS – Congeneric	30816.56 (2)	<0.001	0.734	0.203	0.373	0.099
Simulated Cutoffs	5.98		0.998	0.994	0.028	0.010
Cohen-PSS – With Random Intercept	0.712 (1)	0.399	1.000	1.001	0	0.002
Simulated Cutoffs	3.94		0.999	0.993	0.034	0.006
Schäfer-PSS – Correlated Factors	0.85 (1)	0.310	1.000	1.001	0	0.004
Cohen-PSS – Correlated Factors (Heywood adjustment)	2.34 (1)	0.020	0.998	0.988	0.035	0.010
Simulated Cutoffs	3.36		0.998	0.986	0.037	0.011

With regard to the Schäfer-PSS, the initial model already showed a very good fit. However, it became apparent that the error variance of Item 5 was negative (*θ* = −0.299, *SE* = 0.200, *p* = 0.134). We identified this as a Heywood case since the error term was not significantly smaller than 0, and accordingly constrained this specific variance to a positive value, which marginally worsened the fit but yielded a valid model. The resultant model fit was however still very good by both conventional fixed standards as well as when comparing to simulated cutoffs. Reliability estimates were mixed, ω_Self-Efficacy_ = 0.79 and ω_Helplessness_ = 0.51. For exploratory purposes, we also tested the Schäfer-PSS in a one-factor random intercept configuration. However, this model exhibited unacceptable model fit, χ^2^(2) = 192.84, *p* < 0.001, *CFI* = 0.873, *TLI* = 0.620, *RMSEA* = 0.199, *SRMR* = 0.138.

### 3.2 Convergent correlations

Regarding the direction and magnitude of the correlations (see Table 3), the expected pattern emerged for the congeneric model of the Cohen-PSS. That is, we found large positive correlations with the PBS, the PHQ, and the GAD, as well as a large negative correlation with the FLZ. In including the method factor for the negative items, the correlational pattern remained largely unchanged. In contrast, the two-factorial Schäfer-PSS evinced a more complex pattern of associations: Whereas the Self-Efficacy subscale correlated moderately positively with the FLZ, and negatively with the symptom and burnout scales, the Helplessness subscale exhibited the same pattern of correlations as the original Cohen-PSS, only with somewhat lower magnitude.

**Table 3:** Latent factor correlations.

	PBS (Burnout)	FLZ (Quality of Life)	PHQ-2 (Depression)	GAD-2 (Anxiety)
1 - Cohen-PSS, Congeneric	0.668	−0.610	0.784	0.779
2 - Cohen-PSS, Random Intercept	0.581	−0.596	0.710	0.693
3 - Schäfer-PSS Self-Efficacy	−0.344	0.472	−0.471	−0.438
4 - Schäfer-PSS Helplessness	0.659	−0.447	0.615	0.657

All correlations are significant at 0.001.

## 4 Discussion

The present study sought to compare two competing ultra-short versions of the Perceived Stress Scale – PSS4, the original version provided by Cohen et al. (1983) and the newly-constructed version by Schäfer et al. (2023). Schäfer had initially constructed their two-factorial version of the PSS to improve upon some perceived shortcomings of the original version. Our findings that were yielded by a thorough investigation within a large, population-representative sample indicate that neither of the two instruments can be recommended completely and without any reservation whatsoever. Specifically, the original Cohen-PSS-4 when modeled in a congeneric design has unacceptable model fit, moderate internal consistency, but very high convergent correlations. Upon introduction of a method factor for acquiescent response style (by means of a random intercept), reliability improved markedly and model fit became near-perfect while retaining its correlational pattern with convergent scales. The Schäfer-PSS-4 had a unique challenge because of a Heywood case (negative measurement error term). However, after remedying this issue, we found a model with very good fit, mixed reliability, but reasonable overlap in terms of its validity. That is, reliability was good for the Self-Efficacy subscale but not good for the Helplessness subscale. The correlational pattern corresponded well to the original PSS-4 scale in the case of the Helplessness subscale, but as for the Self-Efficacy scale, associations were in the opposite direction (as expected) but of reduced magnitude. The Schäfer-PSS did not fit well with a one-factor random intercept model, providing evidence for its multi-dimensional structure.

In practical terms, the original Cohen version of the PSS-4 allows for a highly reliable and valid measurement of stress, but not if one relies on the observed score to conduct one’s research. As recent contributions in psychological methods research have pointed out, there can be a big difference between using observed and latent scale scores (McNeish and Wolf, 2020; Schmalbach et al., 2024). This needs to be kept in mind when utilizing the original PSS-4. This is further complicated by the presence of response biases such as acquiescence (Podsakoff et al., 2003). A common and effective remedy is the introduction of a method factor to account for these non-content-related portions of variance (Maydeu-Olivares and Coffman, 2006; Schmalbach et al., 2021). The Schäfer-PSS, or PSS-2&2, on the other hand, is clearly two-dimensional in terms of its content which may be of interest to researchers seeking to differentiate various facets of stress. Because of its uncomplicated design (not including any negative items) it does not suffer from the same method effect issues as the Cohen-PSS. This means that it is more readily interpretable in observed score form. However, it should be noted that it does not provide a total score (unlike the Cohen-PSS), but only facet scores. In addition, the low reliability of the Helplessness scale calls into question how accurate the measurement for this facet actually is. To be fair, it should also be mentioned that Schäfer et al. (2023) found an *ω* of 0.85 for the same scale in their sample. Thus, the scale may very well prove reliable enough in future studies. Finally, the divergence in terms of dimensionality between the PSS-4 and PSS-2&2 indicates that the exact dimensionality of stress and in particular the PSS might need more study.

## 5 Conclusion

Our analyses show that, overall, both ultrashort versions of the Perceived Stress Scale (PSS-4) can be considered reliable and valid – but not without reservation. Our findings emphasize the need to utilize appropriate measurement models for both the psychometric evaluation of an instrument as well as its application in subsequent research questions. The insights gained from this investigation not only contribute to the ongoing refinement of stress measurement instruments but also provide a critical foundation for future research aiming to enhance the precision of stress-related outcomes in diverse populations. As stress remains a pivotal determinant of health, advancing epidemiological as well as clinical research is contingent on the accuracy and reliability of the measurement of the constructs of interest.

## Data Availability

The raw data supporting the conclusions of this article will be made available by the authors, without undue reservation.
