# Supplementary material for: Psychometric comparison of two short versions of the Perceived Stress Scale (PSS-4) in a representative sample of the German population
Source: Front Psychol. 2025 Jan 6;15:1479701. doi: 10.3389/fpsyg.2024.1479701 (PMC11742941; doi:10.3389/fpsyg.2024.1479701)
Supplement: Supplementary file 1 [file Table_1.DOCX]

Supplementary Material

# Supplementary Figures and Tables

Table S1

*Items of the Perceived Stress Scale 10*

| Item | Facet | English version | German version | *M* | *SD* | Skewness | Kurtosis | Cohen PSS-4 | Schäfer PSS-2&2 |
| --- | --- | --- | --- | --- | --- | --- | --- | --- | --- |
| 1 | Helplessness | In the last month, how often have you been upset because of something that happened unexpectedly? | Wie oft waren Sie im letzten Monat aufgewühlt, weil etwas unerwartet passiert ist? | 2.19 | 0.93 | 0.39 | 2.59 |  | X |
| 2 | Helplessness | In the last month, how often have you felt that you were unable to control the important things in your life? | Wie oft hatten Sie im letzten Monat das Gefühl, nicht in der Lage zu sein, die wichtigen Dinge in Ihrem Leben kontrollieren zu können? | 1.89 | 0.94 | 0.88 | 3.20 | X |  |
| 3 | Helplessness | In the last month, how often have you felt nervous and “stressed”? | Wie oft haben Sie sich im letzten Monat nervös und gestresst gefühlt? | 2.42 | 1.00 | 0.35 | 2.71 |  |  |
| 4 | Self-Efficacy | In the last month, how often have you felt confident about your ability to handle your personal problems? | Wie oft waren Sie im letzten Monat zuversichtlich, dass Sie fähig sind, Ihre persönlichen Probleme zu bewältigen? | 3.61 | 1.10 | -0.73 | 2.96 | X | X |
| 5 | Self-Efficacy | In the last month, how often have you felt that things were going your way? | Wie oft hatten Sie im letzten Monat das Gefühl, dass sich die Dinge zu Ihren Gunsten entwickeln? | 3.47 | 1.02 | -0.59 | 2.97 | X | X |
| 6 | Helplessness | In the last month, how often have you found that you could not cope with all the things that you had to do? | Wie oft hatten Sie im letzten Monat den Eindruck, nicht all Ihren anstehenden Aufgaben gewachsen zu sein? | 2.02 | 0.93 | 0.69 | 2.92 |  |  |
| 7 | Self-Efficacy | In the last month, how often have you been able to control irritations in your life? | Wie oft waren Sie im letzten Monat in der Lage, ärgerliche Situationen in Ihrem Leben zu beeinflussen? | 3.59 | 1.07 | -0.65 | 2.94 |  |  |
| 8 | Self-Efficacy | In the last month, how often have you felt that you were on top of things? | Wie oft hatten Sie im letzten Monat das Gefühl, alles im Griff zu haben? | 3.84 | 0.97 | -0.87 | 3.64 |  |  |
| 9 | Helplessness | In the last month, how often have you been angered because of things that were outside of your control? | Wie oft haben Sie sich im letzten Monat über Dinge geärgert, über die Sie keine Kontrolle hatten? | 2.60 | 1.01 | 0.21 | 2.62 |  | X |
| 10 | Helplessness | In the last month, how often have you felt difficulties were piling up so high that you could not overcome them? | Wie oft hatten Sie im letzten Monat das Gefühl, dass sich so viele Schwierigkeiten angehäuft haben, dass Sie diese nicht überwinden konnten? | 2.00 | 0.99 | 0.78 | 3.04 | X |  |

*Note.* The PSS is scored on a 5-point Liker scale (Never, Almost Never, Sometimes, Fairly Often, Very Often / Nie, Fast nie, Manchmal, Ziemlich oft, Sehr oft)
